# To be or not to PHP.eB? Potential strategies for therapeutically “attacking” multiple sclerosis

**DOI:** 10.1016/j.omtm.2025.101574

**Published:** 2025-09-01

**Authors:** Jessica A. Herstine, Benjamin L.L. Clayton

**Affiliations:** 1 Jerry R. Mendell Center for Gene Therapy, The Abigail Wexner Research Institute, Nationwide Children’s Hospital, Columbus, OH, USA; 2 Department of Pediatrics, The Ohio State University Wexner Medical Center, Columbus, OH, USA; 3 Institute for Glial Sciences, Department of Genetics and Genome Sciences, Case Western Reserve University School of Medicine, Cleveland, OH, USA

## Main text

Multiple sclerosis (MS) is a chronic central nervous system (CNS) disease that affects 2.3 million individuals worldwide. Currently, disease-modifying therapies exist, but none fully correct disease, nor target the disease-causing CNS cell types.^1^ In this issue of *Molecular Therapy Methods & Clinical Development*, Nijhuis et al. aim to develop a minimally invasive and comprehensive CNS-directed gene therapy for MS.^2^ To do this, they intravenously (i.v.) deliver a 1:1 mixture of either CNS-specific or ubiquitous adeno-associated virus (AAV).PHP.eB luciferase and EGFP vectors to investigate cellular targeting and distribution in normal and experimental autoimmune encephalitis (EAE) mice, a model of inflammatory demyelination that mimics aspects of MS in humans. Overall, their findings support intravenous administration of the AAV.PHP.eB vectors can achieve CNS-directed expression based on promoter sequence in EAE mice. Direct clinical applicability, however, is unclear since the AAV.PHP.eB capsid is limited in transduction capabilities by species and administration route.^3^^,^^4^^,^^5^ Therefore, future studies will have to be meticulously designed not only by promoter but by capsid as well to allow for human translation.

MS is caused by immune-mediated attacks on myelin, which ultimately results in neuroinflammation and demyelination, and leads to limb weakening, movement difficulties, as well as vision, bladder, bowel, and cognitive dysfunctions.^1^ MS disease progression often starts with periods of relapse and remittance where lesions form and then *mostly* disappear. Over time, however, these lesions fail to repair leading to brain atrophy due to neuronal damage and axonal loss resulting in a progressive chronic disease and neurologic decline.^1^ While no cure exists, current disease modifying therapies that mitigate lesion formation caused by peripheral immune cells are available. These include infusion of interferons or monoclonal antibodies as well as the use of corticosteroids to limit general inflammation.^6^ Indeed, while there are many therapies used in individuals with relapsing-remitting MS, only ocrelizumab, a CD20^+^ B cell targeting monoclonal antibody therapy, is approved for the treatment of progressive MS.^6^ Still, current disease modifying therapies do not address endogenous CNS cell types such as astrocytes and microglia that play a pivotal role in disease progression. Microglia produce and secrete neuroinflammatory cytokines, phagocytic factors, and induce neurotoxic astrocytic phenotypes.^7^ Astrocytes also secrete factors that break down the blood brain barrier (BBB) and recruit more demyelinating peripheral immune cells as well as prevent oligodendrocyte precursors from differentiating into myelin-producing oligodendrocytes.^7^^,^^8^ Finally, oligodendrocytes and neurons get caught in the crossfire, and suffer mitochondrial and oxidative injury ultimately leading to cell death.^9^ Therefore, targeting these cell types to ameliorate neurodegeneration and promote regeneration may prove to be therapeutically beneficial.

One approach to do this is through CNS-directed gene therapy, which delivers genetic material to the brain and spinal cord to prevent disease progression. Using the nonpathogenic, low integrating AAV, therapeutic genes can be introduced to support cellular function and modify disease course. In fact, AAV-mediated gene therapy has been used in over 300 clinical trials and is FDA-approved for the neurologic disease, spinal muscular atrophy.^10^ In the context of MS, CNS-targeted gene therapy is an attractive and underexplored approach to stimulate remyelination, mitigate harmful glia, promote neuroprotection, and potentially slow or halt the shift from relapsing-remitting to chronic progressive forms of the disease.

To support the development of an MS-specific gene therapy, Nijhuis et al. emphasize systemic delivery is the most applicable for MS treatment, as lesion formation is unpredictable and usually surrounds blood vessels. Indeed, generalized administration can broaden—depending on the serotype used—CNS transduction, but increases the risk for off-targeting, toxicity, and the need for larger doses. Thus, to increase gene expression in the brain while limiting toxicity, Nijhuis et al. evaluate CNS-specific or ubiquitous AAV.PHP.eB luciferase and EGFP vectors in both wild-type and EAE mice. By in-life bioluminescence whole body imaging, they confirm the ubiquitous CAG (chicken β-actin with cytomegalovirus early enhancer and a β-globin intron) promoter expresses in the CNS and periphery, while the neural-specific promoters drive expression mainly in the CNS. Further immunohistochemical analysis supports that CAG drives expression in both neurons and astrocytes. Cellular specificity was maintained with their CNS-tropic promoters, with myelin basic protein (MBP) and SRY-box containing gene 10 (SOX10) promoters targeting oligodendrocytes, human synapsin 1 targeting neurons, and glial fibrillary acidic protein (gfa2; also called GFAP), and its truncated version (gfaABC(1)D) targeting astrocytes. Important for enhancing safety for systemic delivery, they demonstrate all neural promoters tested, except for gfa2, restrict expression in the liver.

When assessing how all promoters, except for gfa2, function in EAE conditions, they find that cellular targeting and expression does not change. This observation is particularly interesting in the context of the gfaABC(1)D promoter. In MS and the EAE model, astrocytes are reactive thereby leading to increased GFAP expression. With GFAP elevated particularly near or in lesions, it would be reasonable to assume there would be increased expression of the GFAP-derived vector at these sites as well. They did not see this however, which suggests the loss of certain regulatory regions in the truncated gfaABC(1)D promoter prevents vector upregulation in neuroinflammation. Nonetheless, their oligodendrocyte-targeting MBP and SOX10 promoters did express well in or near lesions, indicating the potential benefit for using these in MS therapeutic vectors.

This study by Nijhuis et al. is an important step forward in the development of CNS-specific gene therapy for MS. Nevertheless, follow-up studies will be needed before this approach is ready for humans. First, targeting glia alone may be limited since they have higher turnover rates leading to episomal washout and waning efficacy. Second, one major MS-causing cell type cannot be targeted with AAV gene therapy—the resident immune cell, microglia. Third, due to the complexity of MS pathology, it may require targeting multiple cell types to have robust therapeutic rescue. Taking these points into consideration, creating one comprehensive, efficacious, and long-lasting gene therapy that minimizes off-targeting will take innovative and fastidious therapeutic design. Fourth, Nijhuis et al. use the PHP.eB capsid due to its better CNS transduction profile in mice as compared to AAV9. Although this is applicable for proof-of-concept experiments, PHP.eB has limited capacity to transduce other animal models.^3^^,^^4^^,^^5^ Thus, another serotype will have to be used in human application, yet this will alter cell specificity, potency, etc. Therefore, additional biodistribution studies may be needed to ensure safe and targeted gene transfer. Finally, serotype choice should consider the progressive breakdown of the BBB as this is key in MS development. Addressing this, Nijhuis et al. rigorously assess Ly6A expression—the receptor used by PHP.eB—in EAE demyelinating lesions. Knowing how disease affects the BBB and using this information for serotype selection will only enhance targetability.

Overall, understanding how we can selectively target distinct populations in the brain and spinal cord is crucial for treating many diseases, including MS. Evaluating expression in the EAE model is the first step to understanding how disease biology affects gene therapy expression. Utilizing those insights will only allow for more innovative, safe, and directed therapeutic approaches to emerge. Although many next steps need to occur to develop, demonstrate, and assess a CNS-targeted gene therapy for MS, the field is moving at an exponential rate. Thus, it may only be a matter of years until we see the insurgence of these types of therapies in clinic, which could only have a huge impact for MS patients.

## Acknowledgments

J.A.H. has received funding from 10.13039/100006928The Ohio State University and 10.13039/100006108NCATS TL1TR002735, T32TR004543, and UM1TR004548. J.A.H. has also received funding from the 10.13039/100000065NINDS F99NS139538. B.L.L.C. has received funding from the 10.13039/100000890National Multiple Sclerosis Society TA-2105-37619 and the 10.13039/100000049NIA P30 AG072959 as a REC Scholar of the Cleveland Alzheimer’s Disease Research Center.

## Declaration of interests

The authors declare no competing interests.
